# Predictors of Dropout in a Digital Intervention for the Prevention and Treatment of Depression in Patients With Chronic Back Pain: Secondary Analysis of Two Randomized Controlled Trials

**DOI:** 10.2196/38261

**Published:** 2022-08-30

**Authors:** Isaac Moshe, Yannik Terhorst, Sarah Paganini, Sandra Schlicker, Laura Pulkki-Råback, Harald Baumeister, Lasse B Sander, David Daniel Ebert

**Affiliations:** 1 Department of Psychology and Logopedics Faculty of Medicine University of Helsinki Helsinki Finland; 2 Department of Clinical Psychology and Psychotherapy Institute of Psychology and Education Ulm University Ulm Germany; 3 Department of Sport Psychology Institute of Sports and Sport Science Albert-Ludwigs-University of Freiburg Freiburg Germany; 4 Clinic for Psychiatry and Psychotherapy Rhein-Erft-Kreis Germany; 5 Medical Psychology and Medical Sociology, Faculty of Medicine University of Freiburg Freiburg Germany; 6 Department for Sport and Health Sciences Chair for Psychology & Digital Mental Health Care Technical University of Munich Munich Germany

**Keywords:** adherence, dropout, law of attrition, attrition, digital health, internet intervention, depression, back pain, comorbidity, mental health, eHealth, mobile phone

## Abstract

**Background:**

Depression is a common comorbid condition in individuals with chronic back pain (CBP), leading to poorer treatment outcomes and increased medical complications. Digital interventions have demonstrated efficacy in the prevention and treatment of depression; however, high dropout rates are a major challenge, particularly in clinical settings.

**Objective:**

This study aims to identify the predictors of dropout in a digital intervention for the treatment and prevention of depression in patients with comorbid CBP. We assessed which participant characteristics may be associated with dropout and whether intervention usage data could help improve the identification of individuals at risk of dropout early on in treatment.

**Methods:**

Data were collected from 2 large-scale randomized controlled trials in which 253 patients with a diagnosis of CBP and major depressive disorder or subclinical depressive symptoms received a digital intervention for depression. In the first analysis, participants’ baseline characteristics were examined as potential predictors of dropout. In the second analysis, we assessed the extent to which dropout could be predicted from a combination of participants’ baseline characteristics and intervention usage variables following the completion of the first module. Dropout was defined as completing <6 modules. Analyses were conducted using logistic regression.

**Results:**

From participants’ baseline characteristics, lower level of education (odds ratio [OR] 3.33, 95% CI 1.51-7.32) and both lower and higher age (a quadratic effect; age: OR 0.62, 95% CI 0.47-0.82, and age^2^: OR 1.55, 95% CI 1.18-2.04) were significantly associated with a higher risk of dropout. In the analysis that aimed to predict dropout following completion of the first module, lower and higher age (age: OR 0.60, 95% CI 0.42-0.85; age^2^: OR 1.59, 95% CI 1.13-2.23), medium versus high social support (OR 3.03, 95% CI 1.25-7.33), and a higher number of days to module completion (OR 1.05, 95% CI 1.02-1.08) predicted a higher risk of dropout, whereas a self-reported negative event in the previous week was associated with a lower risk of dropout (OR 0.24, 95% CI 0.08-0.69). A model that combined baseline characteristics and intervention usage data generated the most accurate predictions (area under the receiver operating curve [AUC]=0.72) and was significantly more accurate than models based on baseline characteristics only (AUC=0.70) or intervention usage data only (AUC=0.61). We found no significant influence of pain, disability, or depression severity on dropout.

**Conclusions:**

Dropout can be predicted by participant baseline variables, and the inclusion of intervention usage variables may improve the prediction of dropout early on in treatment. Being able to identify individuals at high risk of dropout from digital health interventions could provide intervention developers and supporting clinicians with the ability to intervene early and prevent dropout from occurring.

## Introduction

### Background

Chronic back pain (CBP) is a major global health concern with lifetime prevalence rates of 60%-70% [1]. CBP is the leading cause of activity limitation and work absenteeism and accounts for the highest number of disability-adjusted life years worldwide [2]. Approximately 1 in 5 adults with CBP are diagnosed with major depression and many more experience subclinical symptoms [3]. In patients with CBP, comorbid depression is often associated with lower treatment adherence, poorer treatment outcomes, increased medical complications, and higher health care use [4-6].

Psychological interventions have been demonstrated to be effective in the treatment of depression for individuals with CBP [7]. Accordingly, cognitive behavioral therapy is now recommended as the first-line treatment in most international guidelines, for example, the National Institute for Health and Care Excellence [8]. However, the ability to access psychotherapy is a significant barrier to treatment. On average, less than 1 in 5 people in high-income countries and less than 1 in 27 people in low- and middle-income countries receive appropriate treatment [9], giving rise to a *treatment gap* in mental health care [10].

Digital interventions, which deliver psychotherapeutic components via a web browser or smartphone app, have been proposed as a scalable, cost-effective way to meet the growing demand for psychological treatment and address many of the challenges associated with accessing traditional face-to-face therapy [11-15]. There is now a large body of evidence supporting the efficacy of digital interventions for the prevention and treatment of depression [16-20], with recent studies providing promising evidence for the treatment of depression in patients with comorbid physical conditions [21-25].

Despite the demonstrated efficacy, adherence to digital interventions remains a major challenge. A recent meta-analysis on digital interventions for depression identified that, on average, little more than half of the participants completed the full intervention and only 25% of the participants completed the full intervention when the intervention was delivered within routine health care settings [16]. The same meta-analysis also identified that completing the full intervention was the strongest predictor of outcomes, underscoring the importance of treatment adherence [26]. Similar rates of intervention dropout have been reported in other studies on digital health interventions [27-30], giving rise to what Eysenbach [31] has referred to as the “law of attrition,” the observation that a substantial proportion of users in eHealth apps will stop using the intervention before completing the full treatment protocol.

Several studies have assessed predictors of dropout in digital mental health interventions [27,32-34]. In a systematic review of internet-based interventions for anxiety and depression, Christensen et al [34] found that higher baseline depression severity and older age were associated with higher rates of intervention dropout. In a meta-analysis of individual patient data, Karyotaki et al [33] found that being male, having a lower education level, younger age, and comorbid anxiety symptoms significantly increased the risk of dropout from self-guided digital interventions for depression.

However, to the best of our knowledge, no research has been published to date that assesses which factors may predict dropout in a digital intervention for depression in patients with comorbid somatic illness. This question may be particularly pertinent for these individuals as chronic pain and depression are often associated with reduced motivation to initiate or complete goal-directed tasks [35,36]. As the treatment schedules of patients with multimorbidities can already be quite demanding [37,38], higher levels of pain disability—or lower confidence in performing activities while in pain (pain self-efficacy)—may therefore influence an individual’s ability to adhere to a digital intervention and thus experience the benefits [39].

Knowing which patient characteristics predict a higher likelihood of dropout may be valuable for identifying individuals in advance of treatment that might benefit from alternative care pathways [33,40]. However, it is unclear how useful the baseline predictors may be once a patient has already started treatment. In most digital health interventions, there is a steady attrition over time, with most users dropping out after completing the first 1 or 2 modules—the “attrition-phase” [31,41,42]. The ability to identify which individuals are at high risk of dropout early on in treatment could be valuable for supporting clinicians, especially within stepped-care models where rapid identification of nonresponders and the speed of providing alternative treatment can have a significant influence on outcomes [43,44].

Although the field is still nascent, there is a wealth of data generated from digital interventions that may aid the prediction of dropout once a patient has started treatment. In the same way that many digital apps outside of health care use *churn-prediction models* to identify if a user is likely to churn (ie, stop using the app as intended), similar models and principles of human-computer interaction may be valuable to predict dropout and maximize adherence within the field of digital health. For example, in a digital intervention for a chronic lifestyle disease, Pedersen et al [45] were able to predict dropout with 89% precision using a model that included the number of messages received from the health coach, 2 weeks of inactivity, and the provider of the intervention as the most significant predictors. In a study of a self-help digital intervention for the treatment of insomnia, Bremer et al [46] identified individuals at risk of dropout with an area under the receiver operating curve (AUC) of 0.719 using a combination of baseline characteristics (eg, self-reported stress levels) and intervention usage data (eg, number of days to complete each module). One of the most significant findings of the study was that the prediction of user dropout was possible early on in the intervention (after completion of the introductory module).

### Objectives

As digital mental health interventions are being increasingly adopted by health care systems worldwide [13,47], the ability to identify patients at risk of dropout may provide valuable information to improve the adherence, and thus effectiveness, of digital interventions. This study aimed to identify the factors that may predict dropout in a digital intervention for depression in individuals with CBP. In particular, we sought to assess which participant characteristics may be associated with a higher risk of dropout and whether intervention usage data could help improve the identification of individuals at risk of dropout early on in treatment.

Drawing from 2 recent studies on a guided digital intervention for the treatment and prevention of depression in individuals with comorbid CBP, we asked the following research questions:

1. Can we predict intervention dropout from participant baseline characteristics? If so, which participant characteristics predict a higher likelihood of dropout?
2. Can we develop an “early warning system” that identifies participants at risk of dropout early on in the intervention? Specifically, how accurately can we predict which participants will drop out after completion of the first module and what is the most accurate model for classifying at-risk individuals?
3. Do predictors of dropout differ between patients clinically diagnosed with major depressive disorder (where the intervention is targeting treatment) and patients with subclinical depressive symptoms (where the intervention is targeting prevention)?

## Methods

### Study Design

This study was a secondary analysis of data from 2 trials that assessed the efficacy of a therapist-guided internet-based intervention for the treatment [48] and prevention [21] of depressive symptoms in patients with comorbid CBP. Both trials were observer-masked, multicenter, pragmatic, and randomized controlled trials with a parallel design. The trials were conducted simultaneously using the same intervention, procedures, and research setting but targeted individuals with different levels of depressive symptomatology (diagnosed depressive disorder of mild to moderate severity in the study by Baumeister et al [48] and subclinical but at least mild levels of depressive symptoms in the study by Sander et al [21]). For the purpose of this study, the trial data were combined. All the participants provided written informed consent.

### Participants

All participants (N=253) assigned to the intervention arms of the primary studies were included in this analysis. The inclusion criteria of the primary studies were as follows: (1) age ≥18 years; (2) presence of depressive symptoms, either reported persistent subthreshold depressive symptoms in the past 3 months or meeting the Diagnostic and Statistical Manual of Mental Disorders, 4th Edition, criteria for a mild to moderate depressive episode or persistent depressive disorder; (3) diagnosed back pain chronicity of at least 6 months; (4) have German language skills; and (5) access to internet and PC. The exclusion criteria were as follows: (1) having ongoing or planned psychotherapy within the forthcoming 3 months, (2) being currently suicidal or having had suicidal attempts within the past 5 years, or (3) having had a severe depressive episode within the past 6 months. In the primary studies, participants were recruited during or following discharge from 1 of the 82 orthopedic clinics across Germany. They were recruited personally by a clinician or on the web using a flyer and information letters distributed by the clinic.

### Intervention

The intervention is a guided internet- and mobile-based intervention for the treatment (eSano BackCare-D [49]) or prevention (eSano BackCare-DP [50]) of depression in patients with comorbid CBP. The content of the intervention is based on cognitive behavioral therapy for depression and includes elements of psychoeducation, social skills, problem-solving, behavioral activation, relaxation, motivation for physical exercises, and psychological pain intervention elements. Modules consist of information provided by text, video, audio, and interactive exercises and include a homework assignment. At the start of each module, participants reported their perceived stress level at the time and whether they had experienced any negative events in the previous 7 days. There are 6 regular modules and 3 optional modules focusing on sleep, partnership and sexuality, and work. Participants were advised to complete 1 session per week. During the intervention, participants were guided by trained and supervised psychologists (e-coaches) who provided written feedback within 48 hours of each completed module and by answering any queries.

### Measures

#### Baseline Measures

In this study, 8 baseline characteristic variables were assessed as potential predictors of dropout. Variables were chosen on the basis of previous research pointing to demonstrated or hypothetical relationships between the predictor variables and intervention adherence or dropout [32-34,45,46,51]. Demographic characteristics included age, sex (male or female), education level (based on the International Standard Classification of Education by UNESCO [52], low: level 1-2, medium: level 3-4, and high: level 5+), marital status (single, in a relationship, or divorced or widowed), and social support (low, medium, or high). Clinical characteristics included depression, as measured by the Hamilton Depression Rating Scale (HAM-D; [53]); pain disability, as measured by the Oswestry Disability Index (ODI; [54]); and pain self-efficacy, as measured by the Pain Self-Efficacy Questionnaire (PSEQ). The process variables included internet affinity, as measured by the Internet Affinity Scale. Further details on all measurements are provided in the original study protocols [49,50].

#### Intervention Usage Measures

Intervention usage measures included both active and passive measures. The active measures were the stress level reported by the participant at the start of each module (“Burden”) and the occurrence of any negative events experienced in the past 7 days, self-reported by the participant at the start of each module (“Negative Events”). Burden was assessed using a Likert scale from 0 to 10, where 0=“not burdened at all” and 10=“extremely burdened.” Negative events were dummy coded as 0=“no negative event in the past week” and 1=“at least one negative event in the past week.” For passive measures, we included the number of days taken to complete each module (“N Days to complete module”) and the number of minutes spent on the web completing each module (“Time spent online completing module”).

### Dropout

Dropout was defined as completing <6 intervention modules, in accordance with the intervention developers [49,50]. It was operationalized as a binary outcome (dropped out or did not drop out).

### Analytic Strategy

#### Predicting Dropout From Participant Baseline Characteristics

To assess whether participants’ baseline characteristics could predict dropout, analyses were conducted using logistic regression in 3 steps. First, we conducted a series of bivariate analyses to assess the odds ratios (ORs) of each baseline variable (bivariate “bivariate model”). Second, we repeated the analyses with all baseline variables simultaneously entered into the binomial model (the “complete model”). Finally, we built a “parsimonious model” in which we excluded nonsignificant predictors with no incremental predictive power from the complete model in a stepwise procedure.

Akaike information criterion (AIC) and Bayesian information criterion (BIC) were used as measures of model fit and for model comparison. For nested models, likelihood ratio tests were used to directly compare whether 2 models were significantly different from one another [55]. Collinearity was assessed using variance inflation factors and tolerance (1/variance inflation factors). The assumption of linearity of the logit (a linear relationship between the predictors and dropout) was assessed for all continuous predictor variables, and any variables found violating the assumption were transformed based on a visual inspection of the plot.

As this was an exploratory study, we did not adjust for multiple testing. The study was not powered for confirmatory analysis of the predictors, and alpha adjustment may have increased the likelihood of type II errors.

#### Predicting Dropout Early on in the Intervention

To assess whether we could identify people at risk of dropout early on in the intervention, we first created a subset of the data available up until the point of module 1 completion (ie, baseline assessment data and intervention data captured until participants had completed the first module). We then compared the performance of three separate logistic regression models using the constrained data set: (1) a model based on participant baseline characteristics only—the “baseline characteristics model,” (2) a model based on intervention usage variables only—the “intervention usage model,” and (3) a model combining all baseline characteristics and intervention usage variables—the “combined model.” The quality of the models was assessed using the area under the receiver operating characteristic curve (AUROC) and related measures of sensitivity and specificity [55]. The optimal threshold for AUROC was determined using Youden J statistic [56].

Sensitivity analyses were conducted to assess whether predictors differed in the prevention and treatment studies. Here, study was included as a dummy-coded variable (0=PROD-BP for the prevention study and 1=WARD-BP for the treatment study) in all parsimonious models, first as an additional predictor to assess for a main effect of study type on dropout and then as an interaction term with other predictors in the model to assess whether the effect of a predictor differed across studies.

To assess whether the number of modules completed influenced the relative risk of dropout, we conducted sensitivity analyses using Cox proportional hazards regression [57]. In this study, we assessed whether significant predictors of dropout differed between the 2 methods. Analyses were conducted according to the procedures outlined by Eysenbach [31]. The number of completed modules was used as a proxy for time. Models were built using the same 3-step procedure outlined above for logistic regression.

Missingness occurred in 111 out of 3084 (3.6%) data points and was assumed to be missing at random, indicating that missingness depended on observed data [58]. To avoid bias introduced by missingness, missing data were imputed using multiple imputation by chained equations [59,60]. Predictors for missing values were selected based on (1) model-induced predictors, (2) predictors based on bivariate correlation, and (3) bivariate correlation with missingness according to the procedures outlined by van Buuren and Groothuis-Oudshoorn [60]. Predictive mean matching was used as the imputation method. The number of imputed data sets was set to 20, and the number of iterations was set to 10. Convergence was visually assessed and confirmed. Regression analysis was performed on each imputed data set, and the results were pooled according to the rules by Rubin [61]. Sensitivity analyses were conducted using observed (nonimputed) data to compare with the results of the complete models using imputed data.

All analyses were conducted in R using R Studio (RStudio, PBC; [62]). The pROC package was used to calculate the AUROC [63]. The Caret package (R Foundation for Statistical Computing) was used to calculate the sensitivity and specificity [64]. The multiple imputation by chained equations (MICE) package was used for multiple imputation and likelihood ratio tests [60].

### Ethics Approval

This study was a secondary of analysis of data from two RCTs—Sander et al [21] and Baumeister et al [48]. In the original studies, all the participants provided written informed consent. The trial in Sander et al [21] was registered at German Clinical Trials Register (DRKS00007960). The trial in Baumeister et al [48] was registered at the World Health Organization International Clinical Trials Registry (DRKS00009272). All procedures were approved by the ethics committee of the Albert Ludwigs University of Freiburg, Germany (REC No. 8022-6-BW-H-2015; No. 297/14_150513 for the WARD-BP trial, EK-297/14_150513 for the PROD-BP study).

## Results

### Descriptive Statistics

Among the 253 participants, 149 (58.9%) were female and 104 (41.1%) were male. The age of participants ranged from 24 to 78 years, with a mean age of 51.1 (SD 8.88) years. Of the 253 participants, 171 (67.6%) reported having a low level of education. Of the 253 participants, 34 (13.4%) were single, 180 (71.1%) were in a relationship or married, and 39 (15.4%) were divorced or separated. The mean depression severity at baseline was 10.3 (SD 5.93), as measured by the HAM-D, and 9.94 (SD 4.41), as measured by the Patient Health Questionnaire-9. The mean level of pain disability was 31.3 (SD 14.7), as measured by the ODI, and the mean level of pain self-efficacy was 34.9 (SD 13.0), as measured by the PSEQ. Table 1 provides a detailed summary of the demographic and clinical characteristics of the participants.

On average, the participants completed 4.65 out of the 6 regular and 3 optional modules (SD 3.48). The participants took an average of 17.64 (SD 19.55) days to complete each module, and the mean time on the web taken to complete a module was 80.26 (SD 136.96) minutes. The mean self-reported burden was 4.55 (SD 1.97), and the mean number of self-reported negative events across the intervention was 0.80 (SD 1.33). Table 2 shows that 45.1% (114/253) of the participants dropped out of the intervention before completing at least six modules. The table also shows that the number of participants completing the modules decreased steadily as the intervention progressed.

**Table 1:** Demographic and clinical characteristics of the participants (N=253).

Variable		Value^a^
**Age (years)**		
	Mean (SD)	51.1 (8.88)
	Median (range)	52 (24-78)
**Sex, n (%)**		
	Male	104 (41.1)
	Female	149 (58.9)
**Education level, n (%)**		
	Low	171 (67.6)
	Medium	45 (17.8)
	High	37 (14.6)
**Marital status, n (%)**		
	Single	34 (13.4)
	In a relationship (including married)	180 (71.1)
	Divorced or separated	39 (15.4)
**Children, n (%)**		
	Yes	200 (79.1)
	No	53 (20.9)
**Social support, n (%)**		
	None	9 (3.6)
	Low	67 (26.5)
	Sufficient	81 (32)
	High	73 (28.9)
	Very high	23 (9.1)
**Internet affinity (IAS^b^)**		
	Mean (SD)	9.33 (4)
	Median (range)	8.5 (5-25)
	Missing, n (%)	1 (0.4)
**HAM-D^c^**		
	Mean (SD)	10.3 (5.93)
	Median (range)	9 (0-30)
	Missing, n (%)	1 (0.4)
**PHQ-9^d^**		
	Mean (SD)	9.94 (4.41)
	Median (range)	10.0 (1-24)
	Missing, n (%)	3 (1.2)
**Pain disability (ODI^e^)**		
	Mean (SD)	31.3 (14.7)
	Median (range)	30.0 (0-72)
	Missing, n (%)	1 (0.4)
**Pain self-efficacy (PSEQ^f^)**		
	Mean (SD)	34.9 (13)
	Median (range)	36 (0-59)
	Missing, n (%)	1 (0.4)
**Dropout, n (%)**		
	No	139 (54.9)
	Yes	114 (45.1)

^a^Values are based on observed data.

^b^IAS: Internet Affinity Scale.

^c^HAM-D: Hamilton Depression Rating Scale.

^d^PHQ-9: Patient Health Questionnaire-9.

^e^ODI: Oswestry Disability Index.

^f^PSEQ: Pain Self-Efficacy Questionnaire.

**Table 2:** Intervention usage data.

Variable		Value
Modules completed, mean (SD)		4.65 (3.48)
**Participants completing modules, n (%)**		
	Module 1	188 (74.31)
	Module 2	174 (68.77)
	Module 3	159 (62.85)
	Module 4	148 (58.5)
	Module 5	136 (53.75)
	Module 6	128 (50.59)
	Module 7	109 (43.08)
	Module 8	71 (28.06)
	Module 9	61 (24.11)
Days to module completion, mean (SD)		17.64 (19.55)
Time spent on the web completing module (minutes), mean (SD)		80.26 (136.96)
Burden, mean (SD)		4.55 (1.97)
Negative events, mean (SD)		0.81 (1.33)
Dropout, n (%)		114 (45.1)

### Predicting Dropout Using Participant Baseline Characteristics

Table 3 displays the performance of the models used to predict dropout based on the participant baseline characteristics. As the Patient Health Questionnaire-9 and PSEQ scores were highly correlated with HAM-D and ODI (*r*=0.63 and *r*=−0.73, respectively) and were not significant in the bivariate analyses, they were not included in the multivariate predictor models to prevent collinearity. The results of the bivariate analysis indicated that a lower level of education was significantly associated with a higher risk of dropout (OR 2.43, 95% CI 1.19-4.97; *P*=.01, whereas higher age predicted a lower risk of dropout (OR 0.97, 95% CI 0.94-0.99; *P*=.02). None of the other potential predictors (sex, social support, internet affinity, baseline depression severity, and baseline pain intensity) were statistically significant at the level of *P*<.05 in the bivariate analysis.

In the complete model, being single was found to be an additional significant predictor of dropout (OR 2.54, 95% CI 1.09-5.90; *P*=.03). When age was added as a quadratic term (age^2^) to the model to account for the nonlinear relationship between age and dropout, we found that both age (OR 0.63, 95% CI 0.47-0.84; *P*<.001) and age^2^ (OR 1.55, 95% CI 1.17 to –2.05; *P*<.001) were significant predictors, such that both lower and higher age were associated with increased risk of dropout.

In the parsimonious model, where predictors were reduced stepwise to relevant predictors only, low education (OR 3.33, 95% CI 1.51-7.32; *P*<.001) and age (OR 0.62, 95% CI 0.47-0.82; *P*<.001 and age^2^: OR 1.55, 95% CI 1.18-2.04; *P*<.001) remained significant predictors of dropout. Marital status, internet affinity, baseline depression severity, and baseline pain intensity were found to be nonsignificant after controlling for the other predictors.

**Table 3:** Predictors of dropout from participant baseline characteristics.

Predictors		Bivariate model^a^			Complete model^a^			Parsimonious model^a^	
		OR^b^ (95% CI)	*P* value	OR (95% CI)		*P* value	OR (95% CI)		*P* value
Age		0.97 (0.94-0.99)	.02	0.63 (0.47-0.84)		<.001	0.62 (0.47-0.82)		<.001
Age^2^		N/A^c^	N/A	1.55 (1.17-2.05)		<.001	1.55 (1.18-2.04)		<.001
Sex (male)		1.60 (0.96-2.66)	.07	1.68 (0.96-2.94)		.07	N/A		N/A
**Marital status**									
	Single vs in a relationship	1.97 (0.93-4.20)	.08	2.54 (1.09-5.90)		.03	N/A		N/A
	Divorced or widowed vs in a relationship	0.54 (0.26-1.14)	.11	0.62 (0.27-1.42)		.25	N/A		N/A
**Education**									
	Low vs medium	2.43 (1.19-4.97)	.01	3.77 (1.68-8.49)		<.001	3.33 (1.51-7.32)		<.001
	High vs medium	1.88 (0.75-4.71)	.18	2.08 (0.74-5.83)		.16	2.08 (0.78-5.57)		.15
**Social support**									
	Low vs high	0.83 (0.45-1.53)	.55	0.83 (0.41-1.69)		.60	N/A		N/A
	Medium vs high	1.60 (0.88-2.90)	.13	1.64 (0.86-3.14)		.13	N/A		N/A
IAS^d^		1.02 (0.96-1.09)	.53	1.02 (0.95-1.10)		.58	N/A		N/A
HAM-D^e^		0.99 (0.95-1.03)	.67	0.98 (0.93-1.03)		.36	N/A		N/A
Pain disability		1.00 (0.98-1.02)	.85	1.00 (0.97-1.02)		.69	N/A		N/A
Pain self-efficacy (PSEQ^f^)		1.00 (0.98-1.02)	.92	N/A		N/A	N/A		N/A
PHQ-9^g^		0.97 (0.91-1.02)	.24	N/A		N/A	N/A		N/A

^a^Models based on imputed data.

^b^OR: odds ratio.

^c^N/A: Not applicable.

^d^IAS: Internet Affinity Scale.

^e^HAM-D: Hamilton Depression Rating Scale.

^f^PSEQ: Pain Self-Efficacy Questionnaire.

^g^PHQ-9: Patient Health Questionnaire-9.

### Predicting Dropout Early on in the Intervention

Tables 4-6 provide a comparison of the models used to predict dropout following the completion of the first module. In the parsimonious model using only participant baseline characteristics, higher and lower age (OR 0.57, 95% CI 0.41-0.79; *P*=.001 and age^2^: OR 1.68, 95% CI 1.22-2.31; *P*=.001) and low education (OR 2.98, 95% CI 1.04-8.56; *P*=.04) were significant predictors of dropout. The AUROC for the model was 0.70, the sensitivity was 68%, and the specificity was 62%.

In the parsimonious model using only intervention usage data, a higher number of days to module completion predicted a higher risk of dropout (OR 1.04, 95% CI 1.01-1.07; *P*=.005), whereas a self-reported negative event in the previous week was associated with a lower risk of dropout (OR 0.30, 95% CI 0.11-0.81; *P*=.02). The AUROC for the model was 0.61, the sensitivity was 56%, and the specificity was 54%.

In the parsimonious model that combined participant baseline characteristics and intervention usage variables as predictors, higher and lower age (OR 0.60, 95% CI 0.42-0.85; *P*=.004 and age^2^: OR 1.59, 95% CI 1.13-2.23; *P*=.008), medium versus high social support (OR 3.03, 95% CI 1.25-7.33; *P*=.02), and a higher number of days to module completion (OR 1.05, 95% CI 1.02-1.08; *P*=.002) all predicted a higher risk of dropout, whereas a self-reported negative event in the previous week was associated with a lower risk of dropout (OR 0.24, 95% CI 0.08-0.69; *P*=.008). The AUROC for the model was 0.72, the sensitivity was 76%, and the specificity was 59%.

As shown in Table 7, a comparison of the parsimonious models based on participant baseline characteristics and intervention usage variables revealed that the model that combined baseline and intervention usage variables was the most accurate in predicting dropout (AIC=198.9; BIC=253.9) and significantly more accurate than the model using participant baseline characteristics only (AIC=212.6; BIC=254.7; *χ*^²^_181_=5.3; *P*=.006).

**Table 4:** Predictors of dropout following module 1 completion—participant baseline characteristics.

Predictors		Bivariate model^a^			Complete model^a^			Parsimonious model^a^	
		OR^b^ (95% CI)	*P* value	OR (95% CI)		*P* value	OR (95% CI)		*P* value
Age		0.96 (0.92-1.00)	.048	0.61 (0.43-0.87)		.006	0.57 (0.41-0.79)		.001
Age^2^		0.97 (0.93-1.01)	.12	1.58 (1.12-2.24)		.01	1.68 (1.22-2.31)		.001
Sex (male)		1.45 (0.75-2.82)	.27	1.37 (0.65-2.92)		.41	N/A^c^		N/A
**Marital status**									
	Single vs in a relationship	1.79 (0.68-4.72)	.24	2.06 (0.67-6.27)		.20	N/A		N/A
	Divorced or widowed vs in a relationship	0.75 (0.30-1.90)	.55	1.01 (0.35-2.93)		.99	N/A		N/A
**Education**									
	Low vs medium	2.17 (0.83-5.68)	.11	3.60 (1.19-10.88)		.02	2.98 (1.04-8.56)		.04
	High vs medium	2.03 (0.61-6.75)	.25	1.77 (0.46-6.80)		.40	2.18 (0.60-7.85)		.23
**Social support**									
	Low vs high	0.84 (0.34-2.07)	.69	0.80 (0.29-2.22)		.66	N/A		N/A
	Medium vs high	2.65 (1.22-5.80)	.01	2.32 (1.00-5.38)		.05	N/A		N/A
IAS^d^		1.02 (0.94-1.11)	.59	1.02 (0.92-1.13)		.72	N/A		N/A
HAM-D^e^		0.99 (0.94-1.05)	.79	1.00 (0.94-1.07)		.98	N/A		N/A
Pain disability		0.99 (0.97-1.01)	.38	0.98 (0.95-1.01)		.28	N/A		N/A
Pain self-efficacy (PSEQ^f^)		1.00 (0.97-1.02)	.97	N/A		N/A	N/A		N/A
PHQ-9^g^		0.97 (0.90-1.04)	.41	N/A		N/A	N/A		N/A

^a^Models based on imputed data.

^b^OR: odds ratio.

^c^N/A: not applicable.

^d^IAS: Internet Affinity Scale.

^e^HAM-D: Hamilton Depression Rating Scale.

^f^PSEQ: Pain Self-Efficacy Questionnaire.

^g^PHQ-9: Patient Health Questionnaire-9.

**Table 5:** Predictors of dropout following module 1 completion—intervention usage variables.

Predictors	Bivariate model^a^		Complete model^a^		Parsimonious model^a^	
	OR^b^ (95% CI)	*P* value	OR (95% CI)	*P* value	OR (95% CI)	*P* value
Number of days to module 1 completion	1.04 (1.01-1.06)	.007	1.04 (1.01-1.07)	.005	1.04 (1.01-1.07)	.005
Negative events	0.34 (0.13-0.87)	.03	0.30 (0.11-0.81)	.02	0.30 (0.11-0.81)	.02
Burden	0.98 (0.84-1.15)	.83	1.01 (0.85-1.19)	.91	N/A^c^	N/A
Time spent on the web completing module 1	1.00 (0.99-1.01)	.81	1.00 (0.99-1.01)	.94	N/A	N/A

^a^Models based on imputed data.

^b^OR: odds ratio.

^c^N/A: not applicable.

**Table 6:** Predictors of dropout following module 1 completion—baseline and intervention usage variables.

Predictors		Complete model^a^		Parsimonious model^a^	
		OR^b^ (95% CI)	*P* value	OR (95% CI)	*P* value
Age		0.54 (0.36-0.80)	.003	0.60 (0.42-0.85)	.004
Age^2^		1.76 (1.19-2.61)	.005	1.59 (1.13-2.23)	.008
Sex (male)		1.53 (0.65-3.59)	.33	N/A^c^	N/A
**Marital status**					
	Single vs in a relationship	1.88 (0.56-6.34)	.31	N/A	N/A
	Divorced or widowed vs in a relationship	1.21 (0.38-3.78)	.75	N/A	N/A
**Education**					
	Low vs medium	3.21 (0.91-11.33)	.07	N/A	N/A
	High vs medium	1.17 (0.26-5.23)	.84	N/A	N/A
**Social support**					
	Low vs high	0.83 (0.27-2.56)	.75	0.92 (0.34-2.51)	.88
	Medium vs high	3.40 (1.33-8.64)	.01	3.03 (1.25-7.33)	.02
IAS^d^		0.99 (0.88-1.11)	.84	N/A	N/A
HAM-D^e^		0.99 (0.92-1.06)	.78	N/A	N/A
Pain disability		0.97 (0.94-1.00)	.08	N/A	N/A
Number of days to module 1 completion		1.05 (1.02-1.08)	.004	1.05 (1.02-1.08)	.002
Negative events		0.22 (0.07-0.68)	.009	0.24 (0.08-0.69)	.008
Burden		0.96 (0.80-1.17)	.71	N/A	N/A
Time spent on the web completing module 1		1.01 (0.99-1.02)	.28	N/A	N/A

^a^Models based on imputed data.

^b^OR: odds ratio.

^c^N/A: not applicable.

^d^IAS: Internet Affinity Scale.

^e^HAM-D: Hamilton Depression Rating Scale.

**Table 7:** Predictors of dropout following completion of module 1: model comparison (models based on imputed data).

Model	AIC^a^	BIC^b^	AUROC^c^	Sensitivity (%)	Specificity (%)
Model 1: baseline variables	212.6	254.7	0.70	68	62
Model 2: intervention variables	207.5	223.7	0.61	56	54
Model 3: baseline+intervention variables	198.9	253.9	0.72	76	59

^a^AIC: Akaike information criterion.

^b^BIC: Bayesian information criterion.

^c^AUROC: area under the receiver operating characteristic curve.

### Sensitivity Analyses

Sensitivity analyses assessing whether findings differed between the treatment (WARD-BP) and prevention (PROD-BP) studies found no significant difference between the two, either in terms of main effect or interaction effects with other predictors. Sensitivity analyses assessing whether findings differed when using Cox proportional hazards regression versus logistic regression found no difference in the significant predictors. Multimedia Appendix 1 presents the results from the Cox regression analyses. Sensitivity analyses assessing whether the results differed between the models using observed data and those using imputed data revealed no difference in the predictors found to be significant. Results from the models using observed data are presented in Multimedia Appendix 2.

## Discussion

### Principal Findings

This study aimed to identify the predictors of treatment dropout in a digital intervention for the treatment and prevention of depression in patients with comorbid CBP. From the participants’ baseline characteristics, we found that a lower education level and lower and higher age (a quadratic effect) predicted a higher risk of dropout. From the intervention usage variables, we found that a higher number of days to module completion predicted a higher risk of dropout, whereas the occurrence of a negative event in the previous week predicted a lower risk of dropout.

Participants with lower education levels were more likely to drop out of treatment, which is consistent with a large body of research on adherence to both digital interventions and face-to-face psychotherapy [33,51,65-67]. This may reflect the fact that these individuals find it harder to comprehend the intervention material or the digital format, and thus, they lose the motivation to continue [30,51]. It is worth noting that 67.5% (171/253) of the participants in this study were classified as having low levels of education. Lower levels of education have also been associated with longer duration or higher occurrence of back pain [68], underscoring the need for additional research on digital interventions for this particular patient group. The finding that both younger and older age predicted higher risk of dropout suggests that the relationship between age and dropout may be more complex than has been previously identified, either owing to the nonlinear relationship between the two or a possible interaction between age and other factors such as computer literacy [33,69]. More pertinently, it points to the challenges of predicting which individuals are likely to drop out of a digital intervention based on baseline characteristics alone [70]. Finally, the finding that neither pain disability nor depression severity levels were associated with an increased risk of dropout is important as it suggests that digital interventions targeting comorbid depression are acceptable for patients with varying levels of pain intensity and depression symptom severity. This is further supported by the fact that we found no significant difference in predictors when the intervention was aimed at prevention and when it was aimed at treatment. Taken together, these findings provide promising evidence that digital interventions may provide a scalable approach for integrating psychological treatment within pain management routines in health care settings.

This study also demonstrated the feasibility of predicting dropout early on in the intervention based on data restricted to the first module and participant baseline characteristics. Our finding that the number of days taken to complete the first module significantly predicted dropout is consistent with the study by Bremer et al [46] that identified the average number of days taken to complete each module as one of the strongest predictors of dropout in a digital intervention for insomnia. This may reflect a number of underlying causes, including challenges interacting with the intervention, low motivation, lack of time, or low perceived value [40]. The finding that a self-reported negative event in the previous week predicted a lower risk of dropout may be because of the fact that experiencing a negative event (or being asked to report on one) provided greater intrinsic motivation to complete the module and is consistent with research demonstrating that some people drop out from an intervention because they no longer feel they need it [30,31,40]. However, it is worth highlighting that the CIs for the predictor were wide, so the results should be interpreted with caution and examined in future studies using larger sample sizes to determine whether the findings replicate. Notwithstanding, this is the first study to identify that a simple 1-item self-report questionnaire may be used to aid the prediction of dropout during a digital intervention, thus highlighting the potential of incorporating such assessments within digital interventions in the future. Interestingly, the relationship between participant education level and dropout was no longer significant in the models that combined baseline characteristics and intervention usage variables (Table 6). This suggests that a patient’s education level may be less important at predicting dropout when including variables that reflect how the patient interacts with the intervention, such as how long it takes them to complete a specific module.

Indeed, a comparison of models using baseline characteristics and intervention usage variables revealed that a model that combined baseline characteristics and intervention usage data generated the most accurate predictions and was significantly more accurate than models based on baseline characteristics only or intervention usage data only. Moreover, in terms of clinical utility, the AUROC of 0.72 and sensitivity of 76% exceeded the accuracy threshold of 65%-70%, at which clinicians reportedly become willing to act on predictions [71]. Implemented within an intervention, dropout risk models such as this could be used to alert supporting clinicians and health care workers when an individual is at high risk of dropout, so that they are able to intervene early and ideally prevent it.

Notwithstanding the above, there is still significant room for improving model performance. In particular, the development of models that are able to predict dropout *before* completion of the first module would be especially valuable as a significant proportion of individuals drop out before then [65,69]. Developing more accurate models will require intervention developers to capture more granular data related to engagement with the intervention, for example, the number and timing of log-ins, interaction with specific components of the modules (eg, homework; [72]), data specifically related to the intervention target (eg, sleep data for an insomnia intervention; [46]), and additional self-report data such as early measures of therapeutic alliance with the coach [40,73]. Armed with comprehensive intervention usage data such as this, researchers will be better positioned to engineer both handcrafted (theory-driven) and automated features and assess their impact on predictive accuracy. Exploring the role of nonlinear machine learning models in improving model performance is also an area that holds potential, as has been demonstrated in several studies comparing the classification performance of machine learning algorithms with logistic regression in the prediction of dropout [45,46].

Finally, as the findings of this study were specific to one intervention, future research would also benefit from assessing whether the predictors found herein are valuable for predicting dropout in other digital interventions, alternative disorders, and different populations. If it is consistently found that there are a set of variables such as “number of days to module completion” that are associated with higher risk of dropout, these predictors may then be used to inform the basis of models for other interventions in the future. In the same way that outcome feedback technology that identifies individuals at risk of deterioration during treatment has been shown to improve eventual treatment outcomes [43,44], dropout warning systems could be used to alert the supporting clinician, guide care pathways (eg, in stepped-care models), or personalize the intervention itself in the case of self-help interventions. Given the high dropout rates found in real-world settings [16], this will become increasingly important as interventions are implemented within public and private health care systems to meet the growing demand for psychotherapy [47].

### Strengths and Limitations

To the best of our knowledge, this is the first study to examine the predictors of dropout in a digital intervention for depression in individuals with a comorbid somatic illness. This is also the first study to compare whether predictors of dropout differ when the intervention is aimed at prevention (in a subclinical population) versus treatment (in a clinically diagnosed population) and using study samples with clinically verified diagnoses at baseline. Finally, in contrast to most studies conducted to date, which have been based on efficacy trials with small sample sizes and convenient samples [33], this analysis was based on data from 2 large-scale effectiveness trials. These trials were conducted within routine health care settings, where dropout rates are typically significantly higher [16], thereby providing high ecological validity.

Despite these strengths, we acknowledge several limitations of this study. First, the analyses were based on data specific to a prevention and treatment version of one intervention and one population, namely, individuals with depressive symptoms and CBP. As such, the predictors we found to be significant and the subsequent accuracy of the classification models may not generalize to other interventions or other populations. For example, dropout has been found to be significantly greater in unguided interventions than in guided interventions, and the mechanisms underlying dropout may differ between the two [33,51]. Future research would, therefore, benefit from assessing whether the predictors found to be significant in this study generalize to other interventions, populations, and settings. Second, as this was an exploratory study, we did not just adjust for multiple testing as alpha adjustment may have increased the likelihood of type II errors. Future research aimed at replicating the current findings in studies that are sufficiently powered for a confirmatory analysis would be valuable. Third, we had only a limited set of data from the intervention available for analysis. Several studies have demonstrated that a number of other variables derived from intervention usage are valuable in the prediction of both adherence and outcomes, including in-depth measures of engagement such as the frequency of log-ins [45] and interactions with specific content formats [72]. Accordingly, there may be other variables with further explanatory power that were not included in our models. The same applies to baseline characteristics, where studies have shown that data obtained from electronic medical records may be used to identify those at risk of dropout during face-to-face therapy [74,75]. Finally, although dropout in this study was operationalized according to the usage intended by the clinicians who developed the intervention [76], it is important to highlight that it is not always necessary for patients to complete the full per-protocol treatment to benefit clinically [30]. In other words, dropout is not always representative of a negative experience [31,40].

### Conclusions

The high dropout rates associated with digital health interventions remain one of the biggest challenges to their successful implementation in real-world health care settings. Being able to identify individuals at high risk of dropout early on in treatment may provide clinicians and intervention developers with a valuable opportunity to intervene early and prevent dropout from occurring. Using a combined set of predictors from patient baseline characteristics and intervention usage data, we were able to identify individuals at risk of dropout early on in a digital intervention for depression in patients with comorbid CBP. Future research should explore ways of improving model accuracy and investigate the feasibility and efficacy of using these models directly within the interventions themselves to improve adherence.

## Appendix

Multimedia Appendix 1 Survival analyses assessing risk of dropout using Cox proportional hazards regression.

Multimedia Appendix 2 Results of the models using observed (nonimputed) data.

## Abbreviations

AIC: Akaike information criterion
AUC: area under the receiver operating curve
AUROC: area under the receiver operating characteristic curve
BIC: Bayesian information criterion
CBP: chronic back pain
HAM-D: Hamilton Depression Rating Scale
MICE: multiple imputation by chained equations
ODI: Oswestry Disability Index
OR: odds ratio
PSEQ: Pain Self-Efficacy Questionnaire

## Data Availability

The data that support the findings of this study are available on reasonable request from HB (harald.baumeister@uni-ulm.de). Data requesters will need to sign a data access agreement. Provision of data is subject to data security regulations. Investigator support depends on the available resources. The statistical code may be made available to an investigator upon reasonable request to the corresponding author.
