# Supplementary material for: Predictors of Dropout in a Digital Intervention for the Prevention and Treatment of Depression in Patients With Chronic Back Pain: Secondary Analysis of Two Randomized Controlled Trials
Source: J Med Internet Res. 2022 Aug 30;24(8):e38261. doi: 10.2196/38261 (PMC9472049; doi:10.2196/38261)
Supplement: Multimedia Appendix 1 [file jmir_v24i8e38261_app1.docx]

**Appendix 1.** Survival Analyses assessing Risk of Dropout using Cox Proportional Hazards Regression

**Table 1**. Predictors of dropout from Baseline Variables - All Participants

|  | | | | | |  |  |  |  |  |  |  |
| --- | --- | --- | --- | --- | --- | --- | --- | --- | --- | --- | --- | --- |
|  |  |  |  |  |  |  |  |  |  |  |  |  |
| **Predictors** | **Bivariate Model** | | |  | **Complete Model** | | |  | **Parsimonious Model** | | |  |
|  | **PH** | **lower  .95** | **upper  .95** | ***p*** | **PH** | **lower  .95** | **upper  .95** | ***p*** | **PH** | **lower  .95** | **upper  .95** | ***p*** |
| Age | 0.97 | 0.95 | 0.99 | 0.010* | 0.79 | 0.69 | 0.90 | <0.001*** | 0.77 | 0.68 | 0.88 | <0.001*** |
| Age^2 | 0.98 | 0.95 | 1.00 | 0.037* | 1.25 | 1.09 | 1.42 | 0.001** | 1.27 | 1.12 | 1.45 | <0.001*** |
| Gender (male) | 1.44 | 1.00 | 2.09 | 0.05 | 1.30 | 0.89 | 1.91 | 0.174 |  |  |  |  |
| Marital status:   Single vs in a relationship | 1.60 | 0.99 | 2.59 | 0.055 | 1.75 | 1.04 | 2.94 | 0.034* |  |  |  |  |
| Divorced/widowed  vs in a relationship | 0.61 | 0.33 | 1.13 | 0.115 | 0.68 | 0.37 | 1.27 | 0.227 |  |  |  |  |
| Education:  Low vs medium | 2.05 | 1.14 | 3.68 | 0.016* | 2.56 | 1.39 | 4.71 | 0.002** | 2.55 | 1.40 | 4.63 | 0.002** |
| High vs medium | 1.65 | 0.79 | 3.43 | 0.016 | 1.64 | 0.78 | 3.46 | 0.194 | 1.74 | 0.83 | 3.63 | 0.142 |
| Social support:  Low vs high | 0.86 | 0.54 | 1.39 | 0.543 | 0.83 | 0.50 | 1.38 | 0.469 |  |  |  |  |
| Medium vs high | 1.28 | 0.84 | 1.96 | 0.255 | 1.24 | 0.80 | 1.92 | 0.343 |  |  |  |  |
| IAS | 1.01 | 0.97 | 1.06 | 0.539 | 1.02 | 0.97 | 1.07 | 0.474 |  |  |  |  |
| HAMD | 0.99 | 0.96 | 1.02 | 0.691 | 0.99 | 0.95 | 1.02 | 0.547 |  |  |  |  |
| Pain Disability (ODI) | 1.00 | 0.99 | 1.01 | 0.999 | 1.00 | 0.99 | 1.02 | 0.808 |  |  |  |  |
| Pain Self-Efficacy (PSEQ) | 1.00 | 0.99 | 1.01 | 0.893 |  |  |  |  |  |  |  |  |
| PHQ-9 | 0.97 | 0.93 | 1.02 | 0.217 |  |  |  |  |  |  |  |  |

PH = proportional hazards, CI = confidence interval, IAS = Internet Affinity Score, HAMD = Hamilton Depression Rating Scale, PHQ-9 = Patient Health Questionnaire

**Table 2.** Predictors of dropout following completion of Module 1

|  | | | | | | |  |  |  |  |  |  |
| --- | --- | --- | --- | --- | --- | --- | --- | --- | --- | --- | --- | --- |
|  |  |  |  |  |  |  |  |  |  |  |  |  |
| **Predictors** | **Bivariate Model** | | |  | **Complete Model** | | |  | **Parsimonious Model** | | |  |
|  | **PH** | **lower  .95** | **upper  .95** | ***p*** | **PH** | **lower  .95** | **upper  .95** | ***p*** | **PH** | **lower  .95** | **upper  .95** | ***p*** |
| Age | 0.96 | 0.93 | 1.00 | 0.03* | 0.72 | 0.57 | 0.91 | 0.007** | 0.75 | 0.61 | 0.92 | 0.007** |
| Age^2 | 0.97 | 0.94 | 1.00 | 0.087 | 1.35 | 1.07 | 1.70 | 0.010* | 1.3 | 1.06 | 1.59 | 0.011* |
| Gender (male) | 1.42 | 0.81 | 2.49 | 0.223 | 1.87 | 0.92 | 3.79 | 0.082 |  |  |  |  |
| Marital status:   Single vs in a relationship | 1.62 | 0.75 | 3.51 | 0.217 | 1.46 | 0.57 | 3.75 | 0.436 |  |  |  |  |
| Divorced/widowed  vs in a relationship | 0.82 | 0.36 | 1.85 | 0.636 | 0.78 | 0.31 | 1.97 | 0.605 |  |  |  |  |
| Education:  Low vs medium | 2.06 | 0.87 | 4.90 | 0.101 | 2.33 | 0.81 | 6.74 | 0.119 |  |  |  |  |
| High vs medium | 1.92 | 0.67 | 5.55 | 0.225 | 1.21 | 0.34 | 4.31 | 0.767 |  |  |  |  |
| Social support:  Low vs high | 0.85 | 0.38 | 1.92 | 0.701 | 1.02 | 0.40 | 2.58 | 0.972 | 0.92 | 0.4 | 2.13 | 0.851 |
| Medium vs high | 2.24 | 1.16 | 4.30 | 0.016* | 2.83 | 1.31 | 6.13 | 0.008* | 2.13 | 1.06 | 4.26 | 0.033* |
| IAS | 1.02 | 0.95 | 1.09 | 0.642 | 0.94 | 0.86 | 1.04 | 0.243 |  |  |  |  |
| HAMD | 0.99 | 0.95 | 1.04 | 0.796 | 1.01 | 0.94 | 1.07 | 0.831 |  |  |  |  |
| Pain Disability (ODI) | 0.99 | 0.97 | 1.01 | 0.343 | 0.99 | 0.96 | 1.02 | 0.426 |  |  |  |  |
| Pain Self-Efficacy (PSEQ) | 1.00 | 0.98 | 1.02 | 0.938 | - | - | - | - |  |  |  |  |
| PHQ-9 | 0.97 | 0.91 | 1.04 | 0.349 | - | - | - | - |  |  |  |  |
| N Days to  Module completion | 1.02 | 1.01 | 1.02 | <0.001 | 1.02 | 1.00 | 1.04 | 0.034* | 1.03 | 1.01 | 1.04 | 0.001** |
| Negative Events | 0.38 | 0.16 | 0.89 | 0.026* | 0.16 | 0.05 | 0.55 | 0.004** | 0.38 | 0.16 | 0.91 | 0.029* |
| Burden | 0.98 | 0.86 | 1.11 | 0.711 | 0.99 | 0.85 | 1.16 | 0.943 |  |  |  |  |
| Module Duration | 1.00 | 0.99 | 1.01 | 0.666 | 1.01 | 0.99 | 1.02 | 0.316 |  |  |  |  |

PH = proportional hazards, CI = confidence interval, IAS = Internet Affinity Score, HAMD = Hamilton Depression Rating Scale, PHQ-9 = Patient Health Questionnaire
