# Supplementary material for: Predictors of Dropout in a Digital Intervention for the Prevention and Treatment of Depression in Patients With Chronic Back Pain: Secondary Analysis of Two Randomized Controlled Trials
Source: J Med Internet Res. 2022 Aug 30;24(8):e38261. doi: 10.2196/38261 (PMC9472049; doi:10.2196/38261)
Supplement: Multimedia Appendix 2 [file jmir_v24i8e38261_app2.docx]

**Appendix 2.** Results of the models using observed (non-imputed) data

**Table 1.** Predictors of dropout from baseline variables using observed data

| Predictors | **Complete Model** | | |  |
| --- | --- | --- | --- | --- |
|  | **OR** | **lower  .95** | **upper  .95** | ***p*** |
| Age | 0.63 | 0.46 | 0.81 | 0.001** |
| Age^2 | 1.55 | 1.20 | 2.11 | 0.002** |
| Gender (male) | 1.61 | 0.92 | 2.84 | 0.096 |
| Marital status:   Single vs in a relationship | 2.46 | 1.06 | 5.90 | 0.039* |
| Divorced/widowed vs in a relationship | 0.63 | 0.27 | 1.42 | 0.273 |
| Education:  Low vs medium | 3.69 | 1.69 | 8.60 | 0.002** |
| High vs medium | 2.07 | 0.75 | 5.88 | 0.162 |
| Social support:  Low vs high | 0.80 | 0.39 | 1.64 | 0.550 |
| Medium vs high | 1.65 | 0.87 | 3.17 | 0.125 |
| IAS | 1.02 | 0.95 | 1.10 | 0.551 |
| HAMD | 0.98 | 0.93 | 1.03 | 0.423 |
| Pain Disability (ODI) | 0.96 | 0.62 | 1.48 | 0.858 |
| OR = odds ratio, CI = confidence interval, IAS = Internet Affinity Score, HAMD = Hamilton Depression Rating Scale | | | | |

**Table 2.** Predictors of dropout from baseline and intervention usage variables using observed data

| **Predictors** | **Complete Model** | | |  |
| --- | --- | --- | --- | --- |
|  | **OR** | **lower  .95** | **upper  .95** | ***p*** |
| Age | 0.59 | 0.37 | 0.87 | 0.013* |
| Age^2 | 1.64 | 1.13 | 2.55 | 0.015* |
| Gender (male) | 1.70 | 0.69 | 4.21 | 0.246 |
| Marital status:   Single vs in a relationship | 1.07 | 0.27 | 3.84 | 0.916 |
| Divorced/widowed vs in a relationship | 0.71 | 0.19 | 2.34 | 0.587 |
| Education:  Low vs medium | 2.63 | 0.77 | 10.70 | 0.142 |
| High vs medium | 1.01 | 0.21 | 5.08 | 0.987 |
| Social support:  Low vs high | 1.01 | 0.31 | 3.19 | 0.980 |
| Medium vs high | 4.21 | 1.61 | 11.80 | 0.004** |
| IAS | 0.95 | 0.84 | 1.07 | 0.388 |
| HAMD | 1.03 | 0.95 | 1.11 | 0.521 |
| Pain Disability (ODI) | 0.97 | 0.94 | 1.01 | 0.158 |
| N Days to Module completion | 1.05 | 1.01 | 1.09 | 0.011* |
| Negative Events | 0.10 | 0.02 | 0.37 | 0.002* |
| Burden | 1.00 | 0.82 | 1.23 | 0.992 |
| Module Duration | 1.01 | 0.99 | 1.02 | 0.483 |
| OR = odds ratio, CI = confidence interval, IAS = Internet Affinity Score, HAMD = Hamilton Depression Rating Scale | | | | |
